# Integration of Clinical Variables for the Prediction of Late Distant Recurrence in Patients With Estrogen Receptor–Positive Breast Cancer Treated With 5 Years of Endocrine Therapy: CTS5

**DOI:** 10.1200/JCO.2017.76.4258

**Published:** 2018-04-20

**Authors:** Mitch Dowsett, Ivana Sestak, Meredith M. Regan, Andrew Dodson, Giuseppe Viale, Beat Thürlimann, Marco Colleoni, Jack Cuzick

**Affiliations:** Mitch Dowsett and Andrew Dodson, Royal Marsden Hospital; Mitch Dowsett, Institute of Cancer Research; Ivana Sestak and Jack Cuzick, Wolfson Institute of Preventive Medicine, Queen Mary University of London, London, United Kingdom; Meredith M. Regan, Dana Farber Cancer Institute and Harvard Medical School, Boston, MA; Giuseppe Viale, University of Milan; Guiseppe Viale and Marco Colleoni, European Institute of Oncology, Milan, Italy; Beat Thürlimann, Kantonsspital St Gallen, St Gallen, and International Breast Cancer Study Group and Swiss Group for Clinical Cancer Research, Berne, Switzerland.

## Abstract

**Purpose:**

Estimating risk of late distant recurrence (DR) is an important goal for managing women with hormone receptor–positive breast cancer after 5 years of endocrine treatment without recurrence. We developed and validated a simple clinicopathologic tool (Clinical Treatment Score post–5 years [CTS5]) to estimate residual risk of DR after 5 years of endocrine treatment.

**Patients and Methods:**

The ATAC (Arimidex, Tamoxifen, Alone or in Combination) data set (N = 4,735) was used to create a prognostic score for post–5-year risk of DR. Validity of CTS5 (ATAC) was tested in the BIG 1-98 data set (N = 6,711). Time to late DR, 5 years after finishing scheduled endocrine therapy, was the primary end point. Cox regression models estimated the prognostic performance of CTS5 (ATAC).

**Results:**

CTS5 (ATAC) was significantly prognostic for late DR in the ATAC cohort (hazard ratio, 2.47; 95% CI, 2.24 to 2.73; *P* < .001) and BIG 1-98 validation cohort (hazard ratio, 2.07; 95% CI, 1.88 to 2.28; *P* < .001). CTS5 (ATAC) risk stratification defined in the training cohort as low (< 5% DR risk, years 5 to 10), intermediate (5% to 10%), or high (> 10%) identified 43% of the validation cohort as low risk, with an observed DR rate of 3.6% (95% CI, 2.7% to 4.9%) during years 5 to 10. From years 5 to 10, 63% of node-negative patients were low risk, with a DR rate of 3.9% (95% CI, 2.9% to 5.3%), and 24% with one to three positive nodes were low risk, with a DR rate of 1.5% (95% CI, 0.5% to 3.8%). A final CTS5 for future use was derived from pooled data from ATAC and BIG 1-98.

**Conclusion:**

CTS5 is a simple tool based on information that is readily available to all clinicians. CTS5 was validated as highly prognostic for late DR in the independent BIG 1-98 study. The final CTS5 algorithm identified 42% of women with < 1% per-year risk of DR who could be advised of the limited potential value of extended endocrine therapy.

## INTRODUCTION

Women with estrogen receptor (ER) –positive primary breast cancer are generally offered adjuvant endocrine therapy for 5 years. More than 50% of recurrences occur after that time, and several studies have indicated that extending treatment beyond 5 years can improve disease outcome.^1-5^ However, this improvement is relatively modest, and extended therapy carries a risk of adverse effects. Few tools have been developed for selecting patients as candidates for extended endocrine therapy or alternatively identifying those who might be spared such therapy. One approach is to identify patients whose risk after 5 years is so low that any benefit would be outweighed by potential adverse effects.

Clinicopathologic parameters such as tumor size, nodal status, and histopathologic grade are routinely used to estimate risk of breast cancer recurrence at diagnosis; we previously reported a clinical treatment score that integrates these factors to estimate prognosis.^6^ Some of these factors have been reported to be associated with risk after 5 years; for example, we found nodal status was a powerful prognostic marker for late recurrence,^7,8^ whereas tumor size and particularly grade were less prognostic after 5 years. Recently, an overview analysis of > 60,000 women with ER-positive disease, who were scheduled to receive 5 years of endocrine therapy and remained disease free at 5 years, reported the subsequent risk of distant recurrence.^9^ Even in patients with T1N0 disease, the estimated risk of distant recurrence between years 5 and 20 was 10% for those with low, 13% for those with intermediate, and 17% for those with high histologic grades, respectively. Although these data unequivocally demonstrate the importance of these clinicopathologic factors, they include studies from 40 years ago, possibly limiting their relevance for contemporary patients with breast cancer. The data were presented largely as categories (eg, T1, T2), limiting precise estimates of risk for individual patients. Lastly, the largely tamoxifen-treated population did not allow assessment of possible differences between tamoxifen and aromatase inhibitors (AIs) with regard to long-term risk.

We aimed to develop and test the validity of a simple prognostic tool to estimate risk of late distant recurrence (Clinical Treatment Score post–5 years [CTS5]) on the basis of clinicopathologic parameters measured in virtually all patients with breast cancer at diagnosis. We used data from the ATAC (Arimidex, Tamoxifen, Alone or in Combination) trial^10^ as the training set and from the BIG (Breast International Group) 1-98 trial as the testing set.^11^

## PATIENTS AND METHODS

### Study Populations

**Fig A1.**
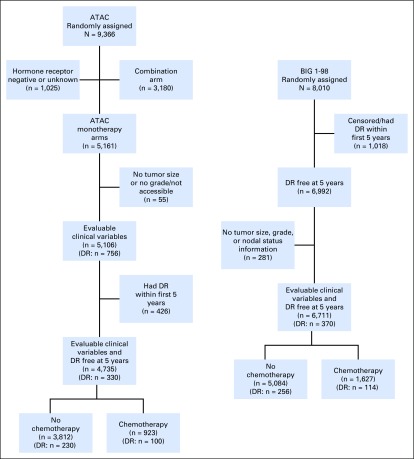
CONSORT diagram according to trial. ATAC, Arimidex, Tamoxifen, Alone or Combination; BIG, Breast International Group; DR, distant recurrence.

CTS5 (ATAC) was trained using data from the ATAC trial (International Standard Randomized Controlled Trial identifier ISRCTN18233230), in which postmenopausal women with ER-positive or ER-unknown early breast cancer were randomly assigned to receive anastrozole 1 mg per day, tamoxifen 20 mg per day, or a combination for 5 years.^10^ The combination arm was discontinued after the first report of trial results.^12^ We included data from women with ER-positive breast cancer randomly assigned to receive anastrozole alone or tamoxifen alone, who were distant recurrence free after 5 years of follow-up and for whom all clinicopathologic data were available (N = 4,735; Appendix Fig A1, online only). Median follow-up was 9.8 years. Data from BIG 1-98 (ClinicalTrials.gov identifier NCT00004205) was used to validate CTS5 (ATAC). BIG 1-98 initially (1998 to 2000) randomly assigned postmenopausal women with hormone receptor–positive early-stage breast cancer to receive 5 years of letrozole 2.5 mg per day or tamoxifen 20 mg per day. Later (1999 to 2003), sequential therapy was also randomly assigned (2 years of letrozole followed by 3 years of tamoxifen or opposite sequence).^11,13^ Median follow-up was 8.1 years. For this analysis, all women were included who were distant recurrence free at 5 years and for whom all clinicopathologic data were available (N = 6,711; Appendix Fig A1). For both trials, women were included in the analysis regardless of whether they received chemotherapy.

Prognostic value of the following variables for post–5-year (late) distant recurrence was determined by univariable Cox regression analyses: nodes, tumor size (in millimeters), grade (1, 2, or 3), age at start of endocrine therapy (years), and type of assigned endocrine treatment. Type of endocrine treatment was not significant for late distant recurrence in univariable analyses and not included in the final model. The log hazard was almost linear for five nodal status groups (negative, one positive, two to three positive, four to nine positive, and > nine positive) but not for continuous tumor size alone. Therefore, a negative quadratic term was introduced, and tumor size was capped at 30 mm, where risk plateaued. The final CTS5 (ATAC) model included age (continuous), tumor size (continuous), quadratic tumor size, nodal status (five groups: 0, negative; 1, one positive; 2, two to three positive; 3, four to nine positive; and 4, > nine positive), and grade (three groups: 1, low; 2, intermediate; and 3, high) and is given by:

CTS5 (ATAC) = 0.471 × nodes + 0.980 × (0.164 × size − 0.003 × size^2^ + 0.312 × grade + 0.03 × age)

A shrinkage factor of 0.980 for the nonnodal part of the score was calculated using a nested Cox model^14^ and applied to allow for the small amount of overfitting. Separate models developed for patients receiving chemotherapy or not did not perform significantly better for either group than a single model including all patients (data not shown).

### Statistical Analyses

Analyses were performed according to a prespecified analysis plan, approved by both trial groups, and are summarized here. Full details are provided in the Appendix (online only). The primary end point was time to distant recurrence, defined as metastatic disease, excluding contralateral disease, and locoregional and ipsilateral recurrences. The end point was censored at last follow-up visit or death before distant recurrence such that risk is a pure risk calculation ignoring deaths.

Cox proportional hazards models were used to create the model in ATAC, and the CTS5 (ATAC) score was tested in BIG 1-98. Likelihood ratio statistics (LR-χ^2^) and Kaplan-Meier survival estimates with corresponding 95% CIs were used to determine the prognostic performance of CTS5 (ATAC) in BIG 1-98. The 5- to 10-year distant recurrence risk groups were determined in ATAC and defined as: low risk, < 5%; intermediate risk, 5% to 10%; and high risk, > 10%. To compare the prognostic performance of CTS5 (ATAC) between ATAC and BIG 1-98 trials, CTS5 (ATAC) was normalized to have unit variance, and hazard ratios (HRs) and associated 95% CIs were estimated from Cox models. All statistical analyses were two sided, and *P* < .05 was regarded as statistically significant. We compared the newly developed CTS5 (ATAC) with the published CTS (termed CTS0 here) developed for estimating prognosis from the time of disease presentation.^6^ All analyses were performed with STATA software (version 13.1; College Station, TX).

## RESULTS

The ATAC training set and the BIG 1-98 test set consisted of 4,735 and 6,711 postmenopausal patients, respectively, assigned to receive 5 years of endocrine therapy (Table 1). Women in the ATAC cohort were significantly older by an average of approximately 3 years and had more node-negative disease (68% *v* 61%) and more grade 3 tumors (25% *v* 20%), and fewer women received adjuvant chemotherapy compared with women in the BIG 1-98 set (19.5% *v* 24.2%). Tumor size was similar between the two trials. In the training set, 330 (7.0%) late distant recurrences were recorded, with an annual hazard rate of 0.79% (95% CI, 0.71% to 0.88%). In BIG 1-98, a total of 370 (5.5%) late distant recurrences occurred, with an annual hazard rate of 0.66% (95% CI, 0.60% to 0.73%), which was significantly lower than in ATAC (*P* = .014; Table 1).

**Table 1.**
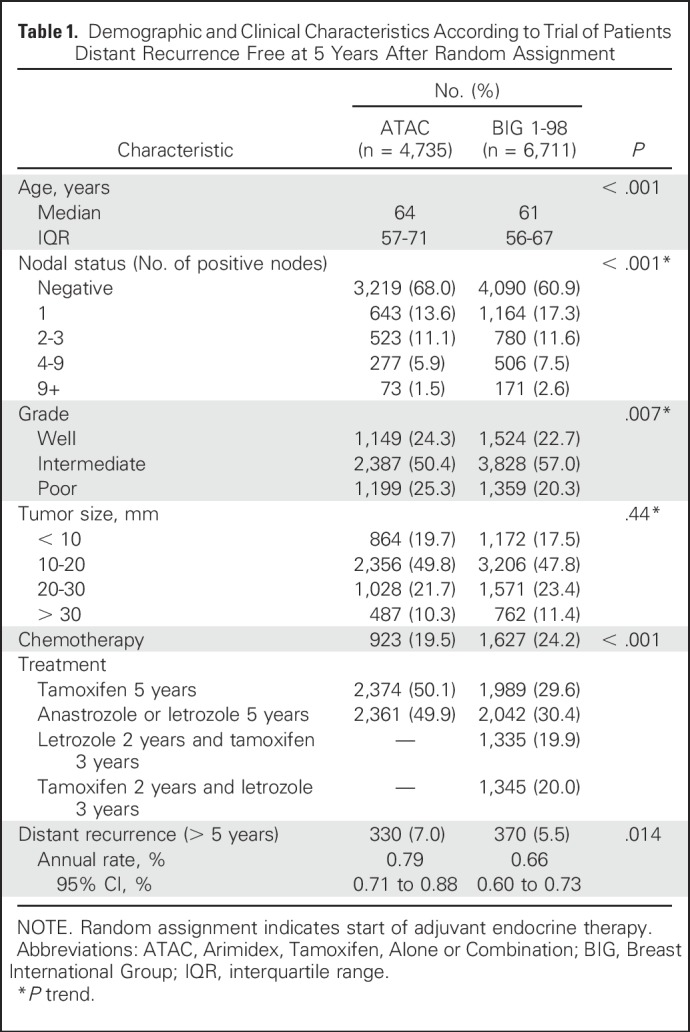
Demographic and Clinical Characteristics According to Trial of Patients Distant Recurrence Free at 5 Years After Random Assignment

### Training Set (ATAC)

Appendix Table A1 (online only) shows the comparisons of the published CTS0^6^ with CTS5 (ATAC) for prediction of late distant recurrence between years 5 and 10. CTS5 (ATAC) provided significantly more prognostic information compared with CTS0 (CTS5 [ATAC]: LR-χ^2^ = 308.6 [5 *df*]; CTS0: LR-χ^2^ = 285.0 [9 *df*]), and larger effect sizes were observed (HR, 2.47 *v* 2.04, respectively). CTS5 (ATAC) was slightly more prognostic in chemotherapy-free women compared with those who received chemotherapy (HR, 2.50; 95% CI, 2.22 to 2.81 *v* 2.39; 95% CI, 1.94 to 2.95), but the interaction with chemotherapy use was not significant (*P* = .76).

**Fig 1.**
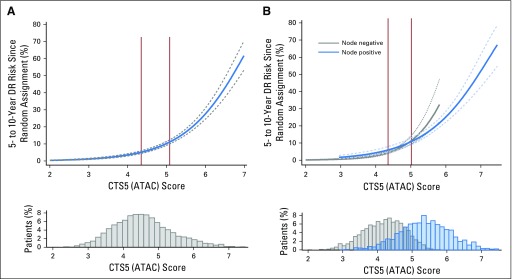
Predicted distant recurrence (DR) risk in years 5 to 10 since random assignment (start of adjuvant endocrine therapy) for ATAC (Arimidex, Tamoxifen, Alone or Combination) trial (A) overall population and (B) node-negative and node-positive patients. Solid vertical lines indicate cutoff points for risk groups. CTS5, Clinical Treatment Score post–5 years.

The prognostic value of CTS5 (ATAC) for risk of distant recurrence (± 95% CI) between years 5 and 10 is shown in Figure 1A for the whole population and in Figure 1B for node-positive and node-negative populations separately. Cutoffs in the ATAC population to separate low-, intermediate-, and high-risk populations were 4.35 and 5.02, respectively (Fig 1A). As expected, most but not all low-risk patients were node negative, and conversely, most high-risk patients were node positive (Fig 1B).

**Fig 2.**
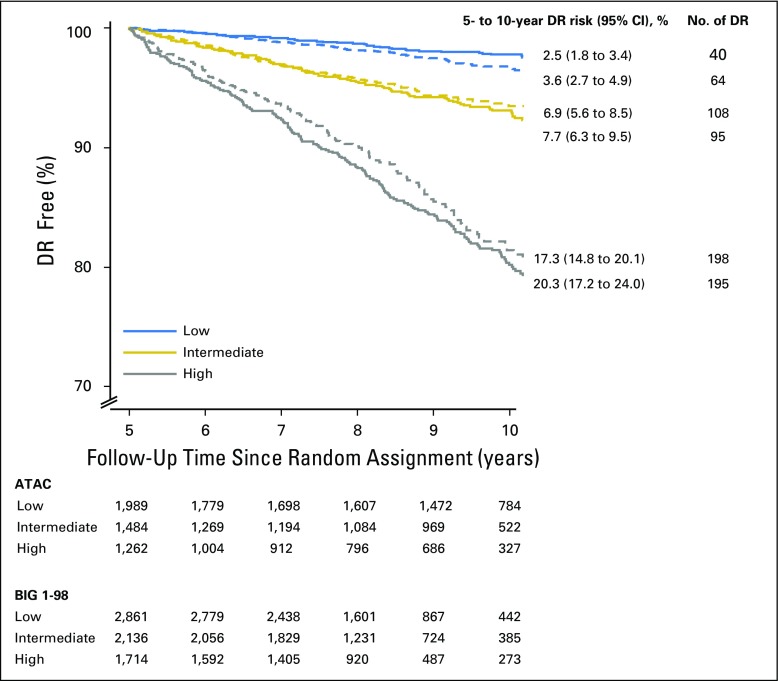
Kaplan-Meier curves and 5- to 10-year distant recurrence (DR) rates since random assignment for the overall population according to trial (solid lines, ATAC [Arimidex, Tamoxifen, Alone or Combination]; dashed lines, BIG [Breast International Group] 1-98).

Overall, 42.0% were categorized as low risk, 31.3% as intermediate risk, and 26.7% as high risk for late distant recurrence (Table 2). Those categorized as low risk had a mean 5- to 10-year distant recurrence risk of 2.5% (95% CI, 1.8% to 3.4%), as compared with 7.7% (95% CI, 6.3% to 9.5%) for intermediate-risk and 20.3% (95% CI, 17.2% to 24.0%) for high-risk groups (Fig 2). Those at intermediate or high risk had a 3.42-fold (95% CI, 2.37- to 4.95-fold) or 9.43-fold (95% CI, 6.71- to 13.25-fold), respectively, higher risk of late distant recurrence than the low-risk group. Notably only two of 133 patients with one to three positive nodes and categorized as low risk had a distant recurrence between years 5 and 10 (Table 2). Virtually all patients with ≥ four positive nodes were categorized as high risk. Approximately one fifth of patients with two or three positive nodes had risk categorized as low or intermediate, whereas 42.9% with one positive node were categorize as high risk. Only 57.7% of node-negative patients were categorized as low risk.

**Table 2.**
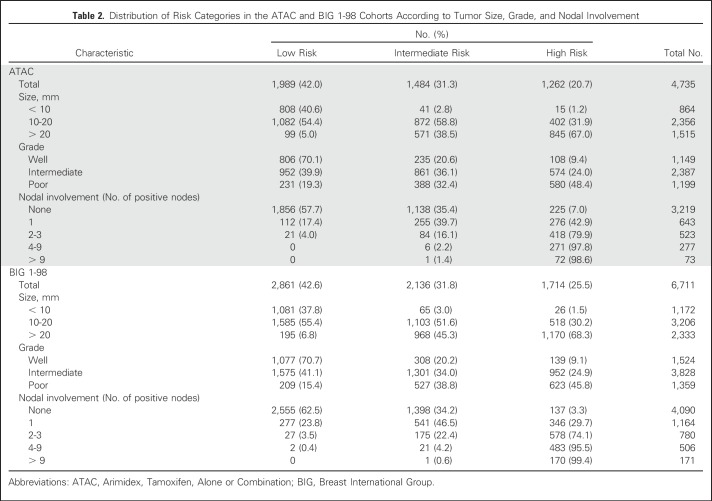
Distribution of Risk Categories in the ATAC and BIG 1-98 Cohorts According to Tumor Size, Grade, and Nodal Involvement

A total of 77 patients experienced local recurrence but no distant recurrence in years 0 to 5, with CTS5 (ATAC) ranking most as intermediate or high risk. Among these 77, CTS5 (ATAC) predicted 24.3 distant recurrences, and 25 were observed. Exclusion of these 77 patients marginally increased the HR for one standard deviation change, from 2.47 (95% CI, 2.24 to 2.73) to 2.53 (95% CI, 2.26 to 2.82).

### Validation Set (BIG 1-98)

CTS5 (ATAC) performed non-significantly better in the validation BIG 1-98 cohort than CTS0 (CTS5 [ATAC]: HR 2.07; 95% CI, 1.88 to 2.28; LR-χ^2^ = 212.1 [1 *df*] *v* CTS0: HR 1.84; 95% CI, 1.70 to 1.98; LR-χ^2^ = 214.9 [1 *df*]). CTS5 (ATAC) was significantly prognostic in women who did not receive chemotherapy (HR, 2.20; 95% CI, 1.96 to 2.47; *P* < .001; LR-χ^2^ = 168.7 [1 *df*]) and more so when compared with those who did (HR, 1.76; 95% CI, 1.46 to 2.13; *P* < .001; LR-χ^2^ = 34.7 [1 *df*]; Appendix Table A1), but the interaction with chemotherapy was not statistically significant (*P* = .06).

**Fig 3.**
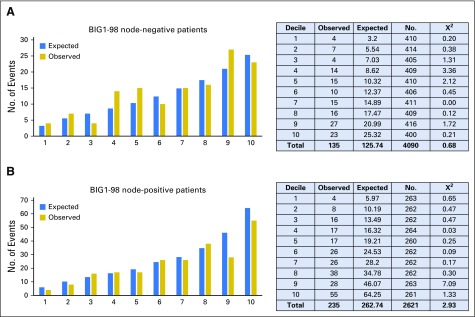
Observed versus expected number of events and χ^2^ values in the BIG (Breast International Group) 1-98 trial according to deciles of Clinical Treatment Score post–5 years (ATAC [Arimidex, Tamoxifen, Alone or Combination]) for (A) node-negative and (B) node-positive patients.

**Fig A2.**
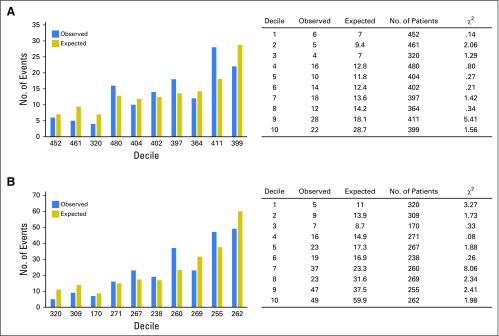
Observed versus expected number of events and χ^2^ values in the BIG (Breast International Group) 1-98 trial according to deciles of published Clinical Treatment Scores (CTSO) for (A) node-negative and (B) node-positive patients. *p* < 0.05 when χ^2^ > 3.84.

The number of observed distant recurrences was compared with those expected by CTS5 (ATAC) in deciles of risk for node-negative and node-positive patients, separately (Figs 3A and 3B). There was a significant difference between the observed and expected numbers for just one of the deciles (9^th^ decile for node-positive). The correlation (*r*) between the observed and expected numbers across the deciles was 0.88 for node-negative and 0.94 for node-positive groups. Using CTS0, a number of deciles showed significant χ^2^ values (Appendix Fig A2, online only), and the *r* values were also lower, at 0.78 and 0.87, respectively. Concordance between the estimated and actual distant recurrence rates was also shown to be better with CTS5 using the Goran-Heller C-index (CTS5 [ATAC], 0.678; CTS0, 0.656).

We used predefined cutoff points of 4.35 and 5.02 from ATAC to determine risk groups for late distant recurrence in BIG 1-98 (Figs 1A and 1B). These cut points intersected the risk curves for BIG 1-98 at 5.4% and 9.9% for node-negative patients and 5.5% and 9.5% for node-positive patients, respectively, and therefore were strongly validated by this test set. The distribution of patients in low-, intermediate-, and high-risk groups was also similar in the BIG 1-98 data set to that observed in the training set (Table 2). The mean 5- to 10-year distant recurrence risk of patients in BIG 1-98 in those three categories was 3.6% (95% CI, 2.7% to 4.9%), 6.9% (95% CI, 5.6% to 8.5%), and 17.3% (95% CI, 14.8% to 20.1%), respectively (Table 2; Fig 2). Thus, for each category, the actual mean risk for each category fitted well with that of the predicted risk. The curves for node-negative and node-positive women were almost identical in the CTS5 (ATAC) regions of overlap in BIG 1-98.

Significant separation between low- versus intermediate-risk groups (HR, 2.19; 95% CI, 1.61 to 2.98) and low- versus high-risk groups (HR, 5.33; 95% CI, 4.02 to 7.07) was observed (Fig 2). Notably, only four of 304 patients with one to three positive nodes and categorized as low risk had a recurrence between years 5 and 10. As with the ATAC data set, in BIG 1-98, virtually all patients with ≥ four positive nodes were categorized as high risk (Table 2). The distribution of patients in the risk categories across histologic grades and nodal categories was similar between ATAC and BIG 1-98. Again, approximately one fifth of patients with two or three positive nodes had risk categorized as low or intermediate, but a somewhat smaller proportion of patients with one positive node were categorize as high risk (29.7% *v* 42.9%). In BIG 1-98, 62.5% of node-negative patients were categorized as low risk, compared with 57.7% in ATAC.

### Combined ATAC and BIG 1-98 Sets

To increase the precision of the risk estimates, we combined the ATAC and BIG 1-98 data sets such that new coefficients were fitted using the same variables as in the training or validation cohort. The final CTS5 is represented by the following model:

CTS5 = 0.438 × nodes + 0.988 × (0.093 × size − 0.001 × size^2^ + 0.375 × grade + 0.017 × age)

**Fig 4.**
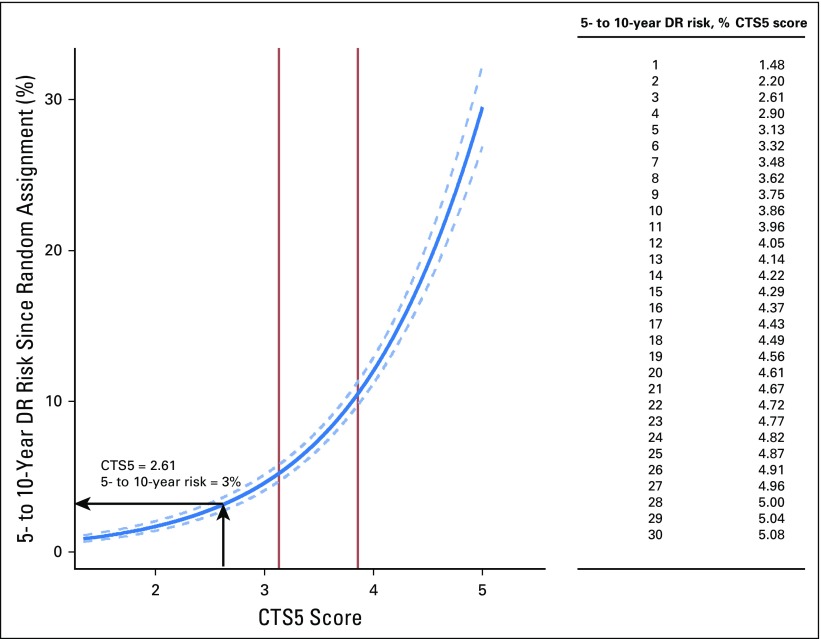
Predicted 5- to 10-year distant recurrence (DR) risk since random assignment and Clinical Treatment Score post–5 years (CTS5) values for the combined data set. Solid vertical lines indicate cutoff points for risk groups. Arrows indicate the CTS5 and equivalent 5- to 10-year risks of a patient age 54 years with a 12-mm, node-negative, grade 2 tumor. Using the formula CTS5 = 0.438 × nodes + 0.988 × (0.093 × size − 0.001 × size2 + 0.375 × grade + 0.017 × age), her CTS5 score is 2.61 and her 5- to 10-year risk of DR is 3%.

The relationship between the final CTS5 and risk of distant recurrence is shown in Figure 4, with a table of CTS5 values that relate to one-unit intervals of distant recurrence risk. New cutoff points for low- (CTS5 < 3.13), intermediate- (3.13 to 3.86), and high-risk (> 3.86) groups were derived from this final model. An example of the calculation of CTS5 and the associated risk estimate is given in Figure 4.

## DISCUSSION

Over the last three decades, there have been major increases in invasive breast cancer incidence in Western countries; in the United States, it was estimated that > 250,000 women would be diagnosed with invasive breast cancer in 2017,^15^ with a large majority of cases localized to the breast. Approximately 80% of patients are now diagnosed as ER positive, and almost all of these are prescribed 5 years of adjuvant endocrine therapy. Although such treatment markedly reduces mortality (eg, by approximately 30% with 5 years of tamoxifen and approximately 40% with an AI in postmenopausal women), recurrences continue to occur after the 5-years treatment ends. The observation that these events can be decreased by continued treatment^1,2,16^ means that decisions about whether to continue with therapy at 5 years are at the forefront of patient management at that time. We expect that the CTS5 tool reported and validated here will prove helpful to oncologists and patients in making a decision about continued treatment. The integration of clinical pathologic features that are measured in all patients at diagnosis should mean that risk is calculable at little expense globally; the table in Figure 4 will allow a direct readout, and an online tool will be provided to facilitate estimates of continuous risk.

Strengths of the study include its use of two large sets of registration-standard randomized clinical trial data with detailed clinical follow-up for 10 years. The ATAC training set included the AI anastrozole as well as tamoxifen as adjuvant treatment, and although the specific endocrine adjuvant therapy did not feature in the algorithm, this allowed us to infer that the score would be valid for both tamoxifen- and AI-treated patients. This is consistent with the overview analysis of AIs versus tamoxifen.^17^ Median 5-year follow-up for the two trials combined occurred approximately 12 years ago. Therefore, it is possible that our risk estimates may not accurately reflect those of current patients reaching 5 years. However, the only major change to the management of primary ER-positive breast cancer since the completion of recruitment to ATAC and BIG 1-98 has been the introduction of trastuzumab for patients with human epidermal growth factor receptor 2–positive disease. CTS5 should be applied with caution in such patients until validated specifically for that population. All patients in the two cohorts were postmenopausal at diagnosis. Although risk of distant recurrence post–5 years has been reported to be similar across age groups, other than for the small group of patients diagnosed at age < 35 years,^9^ the present algorithm cannot be extended to premenopausal patients without further validation.

Neither trial collected complete information on the use of extended adjuvant endocrine therapy. However, the first significant data supporting the use of an AI after tamoxifen^1^ emerged close to the end of the treatment period for the trials, and we estimate that < 1% of tamoxifen-treated patients in ATAC and < 5% in BIG 1-98 received such extended therapy. This would be expected to have minimal impact on our estimates of risk when extended therapy is not used.

Also similar to the report by the Early Breast Cancer Trialists’ Collaborative Group, we found that whether patients had received chemotherapy at presentation had no significant impact on residual risk of recurrence when taking the other factors into account. This may relate in part to the observation that the bulk of the benefit from adjuvant chemotherapy is shown over the first 5 years of follow-up.^18^

The categories of low, intermediate, and high risk were chosen to closely parallel those defined by several molecular profiling tools for managing patients with ER-positive breast cancer.^19-21^ However, those tools are applied immediately after surgery, largely in making the decision of whether to administer adjuvant chemotherapy; what is considered low or high risk in that setting may not be the same when considering the appropriateness of extended adjuvant therapy. In discussions with individual patients whose preferences for continuing or ceasing endocrine therapy at 5 years are likely to vary markedly, the use of a continuous risk estimate from CTS5 is likely to be more informative than the categorical estimates (ie, low, intermediate, and high) used here for illustrative and comparative purposes.

The agreement between the ATAC and BIG1-98 data was almost complete within the low- and intermediate-risk categories but somewhat less beyond the intermediate/high cutoff. Thus, the instrument may be used with greatest confidence in defining 5- to 10-year distant recurrence risk when < 10% and will be of greatest use in assessing the potential value of extended therapy on the basis of risk estimates below that level.

Our report deals only with clinicopathologic profiles. Multigene expression profiles have significantly increased the ability to predict distant recurrence over 10 years after diagnosis in ER-positive breast cancer.^22^ Several of these signatures, such as the Oncotype Dx recurrence score,^23^ PAM50-based Prosigna risk of recurrence score,^19,24^ Breast Cancer Index,^25,26^ EndoPredict test,^20,27,28^ and Netherlands Cancer Institute 70-gene signature,^29^ are commercially available and endorsed by several guidelines.^30-33^ Although a number of them estimate risk of late as well as early recurrence, these tests were developed to manage patients with breast cancer at diagnosis and have not been calibrated for application 5 years after diagnosis. Over the first 10 years of follow-up, clinicopathologic and molecular factors have nearly completely independent prognostic value, and their optimal use for prognosis requires their integration.^34^ It is near certain that the same is true for the 5- to 10-year period. CTS5 provides a straightforward starting point for combining with molecular scores.

## Figures and Tables

**Table A1.**
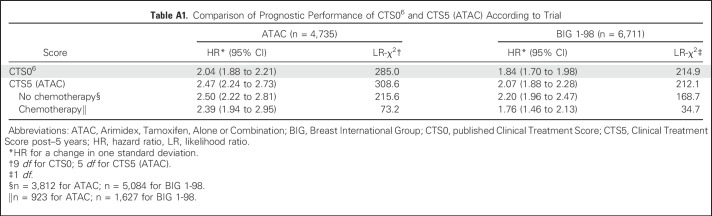
Comparison of Prognostic Performance of CTS0^6^ and CTS5 (ATAC) According to Trial

## Appendix

The primary end point was time to distant recurrence (DR). DR was defined as metastatic disease, excluding contralateral disease, and locoregional and ipsilateral recurrences. The end point was censored at last follow-up visit or death before DR such that risk is a pure risk calculation ignoring deaths.

Cox proportional hazards models were used to create the model in ATAC (Arimidex Tamoxifen Alone or Combination), and the Clinical Treatment Score post–5 years (CTS5; ATAC) was tested in BIG (Breast International Group) 1-98. A shrinkage factor of 0.980 for the nonnodal part of the CTS5 (ATAC) score had been calculated during its derivation using a nested Cox model^14^and applied to allow for the small amount of overfitting. We estimated the shrinkage factor with the following equation:

γ = ([model χ^2^ − *df*]/model χ^2^)^1/2^where model χ^2^ is the likelihood ratio χ^2^ statistic for testing all predictors, and *df* is degree of freedom.

To define the relation between CTS5 and 5- to 10-year DR risk, the logarithm of the baseline cumulative hazard function was fitted. Baseline risk at 5 years was calculated using the stcox/basesurv command in STATA (College Station, TX) to implement the Breslow method. Five- to 10-year DR risk was then calculated for each participant by adjusting the baseline risk:

risk(5-10 years) = 1 − ([baseline risk]^linear prediction CTS5^)

Proportional assumptions were verified using Schoenfeld residuals.

Likelihood ratio χ^2^ statistics and Kaplan-Meier survival estimates with corresponding 95% CIs (calculated from the standard error of the cumulative hazards on the basis of a normal approximation) were used to determine the prognostic performance of CTS5 (ATAC) in BIG 1-98. The risk of DR of events for individual patients in BIG 1-98 was estimated using CTS5 or the published Clinical Treatment Score (CTS0) and the expected risk compared with the observed events in deciles of expected risk. The observed and expected numbers were compared by the χ^2^ test. Overall agreement was assessed by calculating the correlation coefficient across the deciles. Concordance between expected and actual outcomes was also calculated by computing the Goran-Heller C-index.

The 5- to 10-year DR risk groups were determined in ATAC and defined as: low-risk group, < 5%; intermediate-risk group, 5%-10%; and high-risk group, > 10%. To compare the prognostic performance of CTS5 (ATAC) between ATAC and BIG 1-98 trials, CTS5 (ATAC) was normalized to have unit variance, and the hazard ratios and associated 95% CIs were estimated from Cox models. All statistical analyses were two sided, and *P* < .05 was regarded as statistically significant. We also compared the newly developed CTS5 (ATAC) with CTS0, which was developed for estimating prognosis from the time of disease presentation^6^ to determine whether an improved prognostication for late DR had been achieved. All analyses were performed with STATA software (version 13.1).

The final model was fitted on the combined ATAC and BIG 1-98 data sets to give an overall calibration of CTS5. Therefore, new coefficients were fitted in the combined data set but using the same variables as in the training or validation cohort (ie, five nodal groups, continuous age, continuous size, and three grade groups).
